# Effect of Decentration, Rotation, and Tilt on Objective Optical Quality of Plate Haptic Toric Intraocular Lenses in the Early Postoperative Period

**DOI:** 10.1167/tvst.13.2.19

**Published:** 2024-02-26

**Authors:** Yingying Hong, Yang Sun, Houyi Liu, Yinghong Ji

**Affiliations:** 1 Eye Institute and Department of Ophthalmology, Eye & ENT Hospital, Fudan University, Shanghai, China; 2 NHC Key Laboratory of Myopia (Fudan University); Key Laboratory of Myopia, Chinese Academy of Medical Sciences, Shanghai, China; 3 Shanghai Key Laboratory of Visual Impairment and Restoration, Shanghai, China

**Keywords:** optical quality, toric intraocular lens misalignment, OPD-scan III, higher order aberration

## Abstract

**Purpose:**

This study aimed to determine the influence of decentration, rotation, and tilt on objective optical quality of plate haptic toric intraocular lenses (tIOLs).

**Methods:**

The area ratio of modulation transfer function (MTF), strehl ratio of point spread function (PSF), and higher order aberrations (HOAs) for 3 mm and 5 mm pupil diameter (PD) were evaluated at postoperative 1 month. The retroillumination images pictured by OPD-scan III were used to quantify the degree of decentration and rotation, whereas the tIOL tilt was directly obtained by the tilt aberration. Patients were separated into two subgroups based on tIOL misalignment cutoff values.

**Results:**

There were 29 eyes (24 patients) in the study. The decentration of more than 0.25 mm did not substantially differ from those less than or equal to 0.25 mm. PSF of 3 mm PD and MTF, intraocular HOAs, and trefoil aberration for 3 mm and 5 mm PD significantly deteriorated with a rotation of more than 3 degrees, whereas only intraocular HOAs for 5 mm PD and coma for 3 mm and 5 mm PD were significantly severe with a tilt of more than 0.1 µm and 0.25 µm in corresponding PD. Furthermore, tIOL rotation and tilt were highly correlated with intraocular trefoil aberration and coma, respectively.

**Conclusions:**

The decentration of the monofocal bitoric IOLs is more tolerant to optical quality degradation after 1 month of surgery but more sensitive to intraocular trefoil aberration caused by rotation and coma aberration induced by tilt.

**Translational Relevance:**

As far as we know, this is the first study to investigate the relationship between the plate haptic bitoric IOL misalignment and objective optical quality measured by OPD-scan III in the real world, which may provide reference information for IOL selection to improve surgical outcomes.

## Introduction

With the continuous improvement of the quality of life, spectacle independence became an important goal of cataract surgery. It is estimated that 35% of the population with corneal astigmatism is ≥ 1 diopter (D),^1^^,^^2^ and 20% to 30% of patients with cataract with pre-operative corneal astigmatism ≥ 1.25 D are hard to be corrected by monofocal intraocular lens (IOLs).^3^ Thus, the toric IOL (tIOL), first introduced by Shimizu et al. in 1994,^4^ provides an alternative method for correcting corneal astigmatism in patients with cataract, especially low and moderate corneal astigmatism. Importantly, with advancing IOL materials, a wide range of tIOLs are available on the market to optimize surgical methods through smaller incisions, etc., which can be classified according to their raw material, IOL design, and cylinder power. The monofocal hydrophilic acrylic bitoric plate haptic IOL, AT TORBI 709 M (bitoric IOL), as one of the most commonly used tIOL available up to 12.0 D cylinder in 0.50 D steps, has been proven to be safe and effective.^5^

Rotation after IOL implantation is the most prevalent complication of tIOLs, more common in the early postoperative period (especially the first month), which may result in dysfunction of astigmatism correction.^6^^,^^7^ Importantly, it has been reported that a 1 degree of a tIOL misalignment may result in an increasing residual cylinder of 3.3% of the initial power,^8^ whereas postoperative residual astigmatism is the major cause of refractive surprising and not obtaining planned emmetropia following cataract surgery. However, decentration and tilt also have detrimental effects on optical quality, except for tIOL rotation.^9^^–^^13^ The Kim et al. study^10^ evaluated the rotation and decentration of four aspheric toric IOLs on the image quality and showed that the decentration and rotation tolerance of different tIOLs were varied. In order to objectively evaluate the effect of rotation on objective image quality of tIOL, Tognetto et al.^9^ creating an experimental optoelectronic test bench showed the decay of image quality related to rotation degree. Toric 709 IOL with plate design has proved to own more rotation stability,^7^ but there are a lack of investigations on the relationship between tilt and decentration with objective quality. In addition, the accuracy and convenience of OPD-scan III as an aberrometer have been proven.^14^

Therefore, the purpose of the study is to research the effect of decentration, rotation, and tilt on the objective quality measured by OPD-scan III to explore the optical properties of this type tIOL and may provide reference information for IOL selection to improve surgical outcomes.

## Methods

### Patient Selection

The study was a retrospective case analysis and collected clinical and follow-up data from 24 patients (29 eyes) who underwent cataract phacoemulsification in combination with AT TORBI 709 MP Toric IOL implantation (Zeiss Meditec AG, Jena, Germany) at the Department of Ophthalmology of Fudan University affiliated Eye, Ear, Nose, and Throat hospital from February 2021 to February 2022. We included patients with visually significant cataracts and regular astigmatism of at least 1.0 D. The following conditions were excluded from the study: irregular astigmatism, uveitis, advanced glaucoma, and extensive fundus lesions. This study followed the principles of the Helsinki Declaration, and a protocol for the study was in accordance with the approval from the Ethics Committee of the Affiliated Eye & ENT Hospital of Fudan University.

### Pre-Operative Examinations

A comprehensive pre-operative ophthalmologic examination was conducted for all enrolled patients, including visual acuity, slit-lamp biomicroscopy, intraocular pressure, corneal endotheloscopy, fundoscopy, B-scan ultrasonography, and optical coherence tomography (OCT). IOL Master 700 (Carl Zeiss Meditec AG, Jena, Germany) was used to perform biological measurements, including axial length, anterior chamber depth, and keratometry, and Pentacam (Oculus Optikgeräte) Scheimpflug tomography was conducted for each case to ensure the regularity of astigmatism. Based on IOL Master biometric data, the spherical cylinder powers, as well as an intended axis for each IOL were calculated by the manufacturer's online calculator.

### Surgical Procedure

All surgeries were operated under the guidance of surgical navigation by one experienced surgeon (author Y. Ji) with a 2.2 mm corneal clear incision. After injection of the ophthalmic viscosurgical device (OVD) into the anterior chamber, a diameter of 5.5 mm of the continuous curvilinear capsulorhexis was made which was well-centered. Hydrodissection was subsequently performed, followed by cataract phacoemulsification, and thorough removal of lens cortex. After injecting OVD within the empty capsular bag, the accurate implantation of tIOL in the capsular bag was guided by the Zeiss Callisto Eye (Carl Zeiss AG, Dublin, CA, USA). It was possible for the surgeon to visualize one or three parallel lines representing the target meridian and rotate the tIOL accordingly. Careful removal of the OVD was performed, and the IOL alignment was checked and confirmed. Finally, all incisions were hydrated, and dexamethasonetobramycin ophthalmic ointment was topically applied to the conjunctival sac of the operated eyes.

### Postoperative Follow-Up

**Figure 1.**
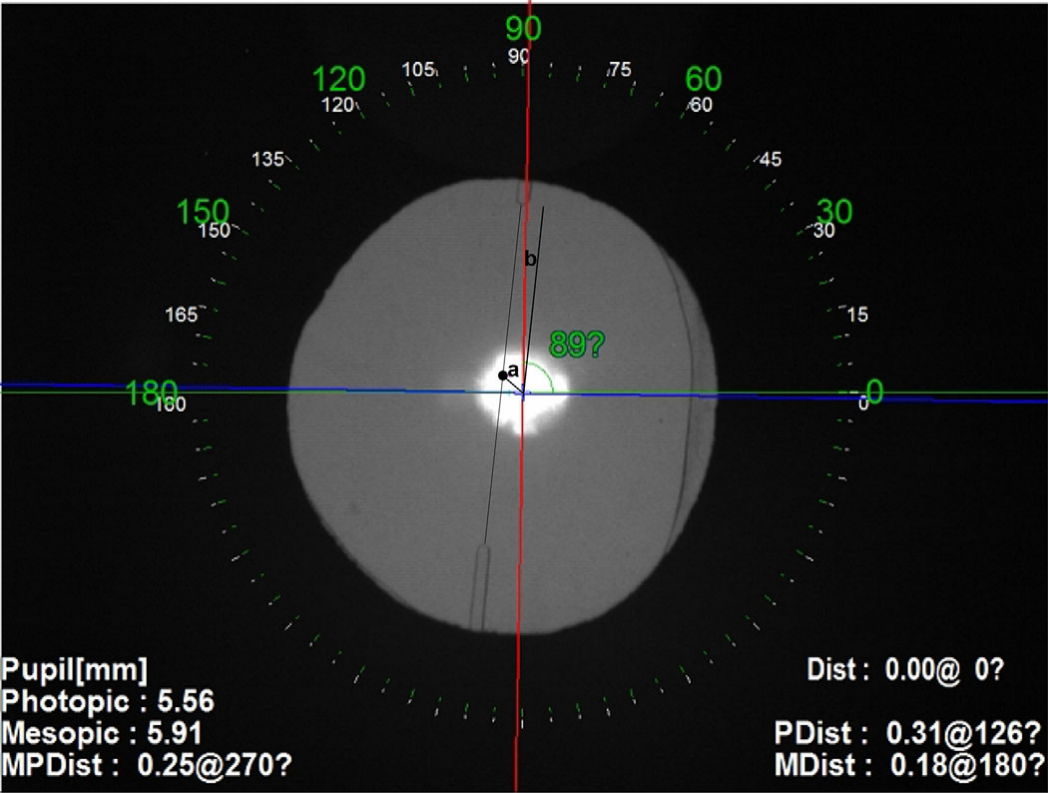
Schematic diagram of tIOL decentration (**a**) and misalignment angle (**b**) measurement method.

The postoperative measurements at 1 month consist of corrected-distance visual acuity recorded and analyzed with logarithm of the minimum angle of resolution (logMAR). The double angle plots of pre-operative corneal astigmatism and postoperative refractive astigmatism were drawn by the Astigmatism Double Angle Plot Tool V132 downloaded from https://ascrs.org/tools/astigmatism-double-angle-plot-tool. After surgery, the exact position of the lens was determined on retroillumination images (Fig. 1) by using the OPD-Scan III (Nidek Inc., Tokyo, Japan) after pupil dilation with a mixture of 0.5% phenylephrine and 0.5% tropicamide (Mydrin-P; Santen Pharmaceutical). Decentration of IOL refers to the distance from the visual axis and the center of the tIOL (the midpoint of the axis marks of IOL on the inside) measured by Adobe Photoshop software (Adobe, San Jose, CA, USA) on the retroillumination images. The misalignment angle of a tIOL refers to the angle between the planned implantation axis of the IOL and the final IOL axis. The tIOL tilt was expressed by the intraocular tilt aberration (µm) for the 3 mm and 5 mm pupil diameter (PD). In order to avoid interference of pupil size on visual quality data, the aberration measurement, including ocular and intraocular higher‑order aberrations (HOAs, including coma, spherical, and trefoil aberration), modulation transfer function (MTF), and point spread function (PSF) were performed by OPD-scan III instrument under 3 mm and 5 mm of pupil diameter as assessment of objective optical quality, and root mean square (RMS) of these values were used for analysis. A measurement of the MTF was provided as the area ratio (AR) between the area under an eye's MTF curve and the area under a perfect optical system's MTF curve, which is a kind indicator for evaluating the visual quality. The Strehl ratio (SR) of PSF was defined as the ratio between the theoretical diffraction limit and the PSF value (range from 0 to 1), and the eye can be regarded as having almost no aberration when this value is 0.8 or larger. To access the impact of IOL decentration, rotation, and tilt on the objective optical quality, we further divided the patients into two subgroups according to the median cutoff value (with decentration of ≤ 0.25 mm or > 0.25 mm; rotation of ≤ 3 degrees mm or > 3 degrees; tilt [3 mm PD] of ≤ 0.1 µm or > 0.1 µm; and tilt [5 mm PD] of ≤ 0.25 µm or > 0.25 µm).

### Statistical Analysis

The statistical analyses were conducted by SPSS statistical software for macOS (version 26, IBM Corp.). Normally distributed data determined by the Shapiro–Wilk test were shown as the mean ± standard deviation (SD). Otherwise, they were shown in median (interquartile range). When the continuous data conformed to a normal distribution, the independent samples *t*-test was performed to compare two groups; otherwise, the nonparametric test was used (Mann–Whitney *U* test). The bar plots of comparison drawn by Prism 9 for macOS (version 9.0.2). When the continuous data conformed to a normal distribution, Pearson's correlation coefficients were conducted to evaluate how objective optical quality parameters were related to overall decentration, rotation, and tilt; otherwise, Spearman's correlation analysis was used. Multiple linear stepwise backward regression analysis was performed to investigate predictors of objective optical quality, including age, axial length, postoperative spherical equivalent (SE), IOL decentration, rotation, and tilt (as independent variables). Differences were considered statistically significant if the *P* values with 2 sides were less than 0.05.

## Results

A total of 29 eyes of 24 patients, with 17 women and 12 men, were analyzed after inclusion and exclusion criteria applied. Detailed demographic information about these patients is presented in Table 1. The mean age was 59.64 ± 11.83 years. The mean AL and ACD were 26.88 ± 2.91 mm and 3.29 ± 0.39 mm, respectively. The median (interquartile range) of IOL cylinder power was +2.5 (+2.5 to +3.5) D.

**Table 1.** Demographic Data of All Patients

Parameters	Value
Patient/eyes no. (right/left)	24/29 (16 to 13)
Sex (female/male)	17/12
Mean ± SD	
Age, y	59.64 ± 11.83
Preoperative corneal astigmatism, D	−2.44 ± 0.68
AL, mm	26.88 ± 2.91
ACD, mm	3.29 ± 0.39
Median (interquartile range)	
IOL cylinder power, D	+2.5 (+2.5 to +3.5)
Preoperative VA (logMAR)	0.60 (0.40 to 0.96)
Postoperative CDVA (logMAR)	0.10 (0 to 0.19)
Postoperative refractive astigmatism	−0.5 (−0.75 to −0.5)

ACD, anterior chamber depth; AL, axial length; CDVA, corrected-distance visual acuity; CTR, capsular tension ring; D, degree; logMAR, logarithm of the minimum angle of resolution; No., number; SD, standard deviation; VA, visual acuity.

Normally distributed data was shown in the mean + standard deviation, and was shown in median (interquartile range) otherwise.

### Astigmatism

**Figure 2.**
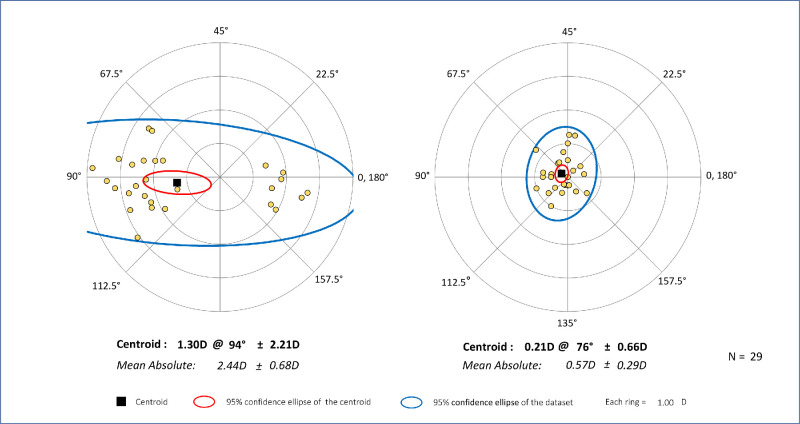
The double angle plots of pre-operative corneal astigmatism and postoperative refractive astigmatism.

Table 2 showed the distribution of eye astigmatism before and after surgery. Compared with preoperative corneal astigmatism, postoperative residual astigmatism was reduced with 90% of eyes within 1.0 D and 100% within 1.5 D. Figure 2 displayed the double-angle plots of astigmatism distribution of included patients. The mean absolute astigmatism from 2.44 ± 0.68 D (1.3 D at 94 degrees ± 2.21 D) significantly decreased to 0.57 ± 0.29 (0.21 D at 76 degrees ± 0.66 D) after tIOL implantation (*P* < 0.001, paired *t*-test). To figure out the influence of cylinder power of tIOL, Table 3 demonstrated that the decentration, rotation, and tilt for 3 mm PD and 5 mm PD of these two groups, divided by the median value +2.5 D (≤ 2.5 D and > 2.5 D) of tIOL cylinder power, were not significant differences (*P* > 0.05).

**Table 2.** Distribution of Eyes Astigmatism Before and After Surgery

	Preoperative Corneal Astigmatism		Residual Astigmatism	
≤ 0.25 D	0	0%	6	21%
≤ 0.50 D	0	0%	18	62%
≤ 0.75 D	0	0%	22	76%
≤ 1.00 D	0	0%	26	90%
≤ 1.50 D	2	7%	29	100%
≤ 2.00 D	10	34%	29	100%

D, Diopters.

**Table 3.** Effect of IOL Cylinder Power on Optical Quality Outcomes (Median, Interquartile Range)

	Total	IOL Cylinder Power ≤ 2.5 D		IOL Cylinder Power > 2.5 D		
Parameters	Median (Interquartile Range)	Median (Interquartile Range)	No.	Median (Interquartile Range)	No.	*P* Value
Decentration	0.26 (0.20 to 0.37)	0.22 (0.16 to 0.37)	15	0.29 (0.23 to 0.37)	13	0.170
Rotation	2.70 degrees (0.80 degrees to 4.50 degrees)	2.40 degrees (0.80 degrees to 4.50 degrees)	15	3.3 degrees (1.10 degrees to 5.85 degrees)	13	0.316
Tilt 3 mm PD	0.09 (0.05 to 0.13)	0.09 (0.05 to 0.13)	16	0.08 (0.04 to 0.13)	13	0.746
Tilt 5 mm PD	0.26 (0.14 to 0.42)	0.33 (0.13 to 0.57)	16	0.26 (0.15 to 0.40)	13	0.559

D, degree; IOL, intraocular lens; PD, pupil diameter; *P* < 0.05.

### Decentration, Rotation, and Tilt Stability of tIOL

As one of the patients was able to obtain 5 mm tilt data, but the axial astigmatism marker of tIOL was not observed on the retroillumination picture, the distance of decentration and rotation of the patient was not available. Overall, the median of the IOL decentration was 0.26 mm (0.20 to 0.37). Two eyes (7.1%, 2/28) had a tIOL decentration of more than 0.5 mm, and 13 eyes (46.4%, 12/28) were less than or equal to 0.25 mm. The median of the IOL rotation was 2.7 degrees (0.8 degrees to 4.5 degrees). There were 13 eyes (46.4%, 13/28) with clockwise rotation and 15 eyes (53.6%, 15/28) with anticlockwise rotation. No eye with tIOL rotation greater than 20 degrees. Additionally, two eyes (7.1%, 2/28) had a tIOL rotation of more than 10 degrees, and 4 eyes (14.3%, 4/28) had a tIOL rotation between 5 degrees and 10 degrees. The median of tIOL tilt for 3 mm PD and 5 mm PD were 0.09 µm (0.05 to 0.13) and 0.26 µm (0.14 to 0.42), respectively. One eye (3.4%, 1/29) of tIOL intraocular tilt for 3 mm PD was greater than 0.5 µm, and 11 eyes (37.9%, 11/29) of tIOL tilts were between 0.1 µm and 0.5 µm. One eye (3.4%, 1/29) of tIOL intraocular tilt for 5 mm PD was greater than 2.0 µm, and 4 eyes (13.8%, 4/29) of tIOL tilts were between 0.5 µm and 1.0 µm.

### Correlation Between tIOL Misalignments and Optical Quality

**Figure 3.**
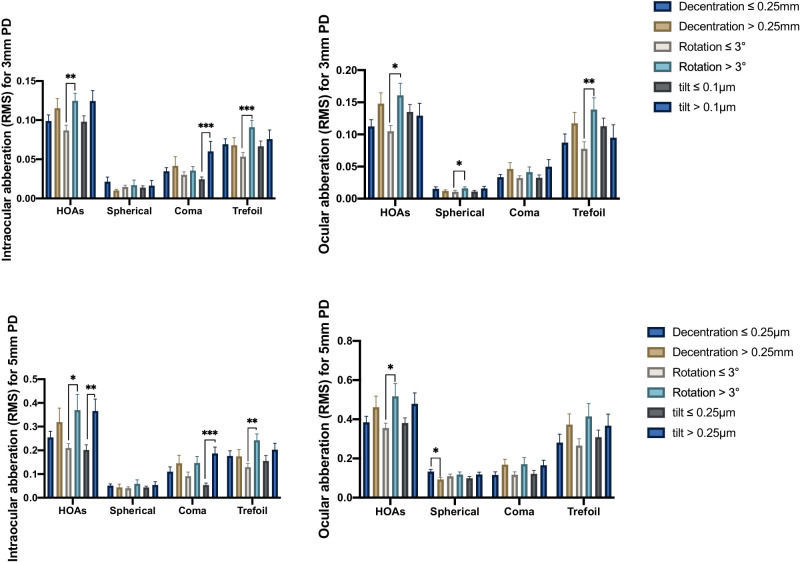
Subgroup comparison of intraocular (*left*) and ocular (*right*) aberrations among at different pupil diameters (PDs). Error bars represent standard errors of the mean. HOAs, higher order aberrations; RMS, root mean square.

To investigate the relationship between tIOL misalignments and objective optical quality, the decentration of 0.25 mm, rotation of 3 degrees, tilt for 3 mm PD of 0.1 µm and tilt for 5 mm PD of 0.25 µm as the cutoff value for grouping and statistical analysis (Table 4, Figure 3). For AR of MTF and SR of PSF (see Table 4), the rotation less than or equal to 3 degrees had a lower AR of MTF (3 mm PD, *P* *=* 0.018; and 5 mm PD, *P* *=* 0.025) and SR of PSF (3 mm PD, *P* *=* 0.008) than rotation greater than 3 degrees, but there were no significant differences between rotation and SR of PSF (5 mm PD) in these 2 groups (*P* *=* 0.223). For tIOL decentration and tilt subgroups, there were no significant differences in AR of MTF and SR of PSF (*P* > 0.05).

**Table 4.** Subgroup Comparisons of Optical Quality Outcomes (Mean ± SD or Median/Interquartile Range)

	Decentration (mm)			Rotation, Degrees			Tilt (µm)					
Parameters	≤ 0.25	> 0.25	*P*	≤ 3 degrees	> 3 degrees	*P*	≤ 0.1 (3 mm PD)	> 0.1 (3 mm PD)	*P*	≤ 0.25 (5 mm PD)	> 0.25 (5 mm PD)	*P*
No.	13	15	—	16	12	—	17	12	—	17	12	—
AR of MTF (3 mm PD) (%)	73.69 ± 16.48	62.33 ± 19.11	0.107^b^	74.62 ± 18.93	58.24 ± 13.67	0.018^a^^,^^b^	65.32 ± 16.72	69.45 ± 21.20	0.562^b^	73.03 ± 16.67	62.15 ± 18.89	0.116^b^
AR of MTF (5 mm PD) (%)	49.91 ± 8.04	50.56 ± 14.56	0.888^b^	54.49 ± 11.62	44.63 ± 9.78	0.025^a^^,b^	49.05 ± 10.23	51.00 ± 14.07	0.668^b^	52.29 ± 9.46	47.88 ± 13.33	0.325^b^
SR of PSF (3 mm PD)	0.38 ± 0.17	0.26 ± 0.17	0.081^b^	0.38 ± 0.19	0.21 ± 0.11	0.008^a^^,b^	0.28 ± 0.16	0.34 ± 0.20	0.396^b^	0.36 ± 0.18	0.27 ± 0.17	0.188^b^
SR of PSF (5 mm PD)	0.036 (0.031, 0.050)	0.043 (0.026, 0.068)	0.812^c^	0.046 (0.032, 0.068)	0.040 (0.027, 0.045)	0.223^c^	0.037 (0.028,0.046)	0.044 (0.028, 0.052)	0.419^c^	0.040 (0.029, 0.049)	0.038 (0.027, 0.050)	0.746^c^

AR, area ratio; MTF, modulation transfer function; No., number; PD, pupil diameter; PSF, point spread function; SD, standard deviation; SR, Strehl ratio.

a Statistically significant (*P* < 0.05).

b Independent samples *t*-test.

c Mann–Whitney *U* test.

There were no significant differences with decentration for HOAs of 3 mm PD and 5 mm PD (see Fig. 3) except for the ocular spherical aberration for 5 mm PD (*P* = 0.015). In the aspect of HOAs and trefoil aberration, the rotation less than or equal to 3 degrees was lower than those of more than 3 degrees, including intraocular and ocular aberration for 3 mm and 5 mm PD (5 mm PD ocular: HOAs: *P* = 0.015; 5 mm PD intraocular: trefoil: *P* = 0.001, HOAs: *P* = 0.010; 3 mm PD ocular: trefoil: *P* = 0.004, HOAs: *P* = 0.017; and 3 mm PD intraocular: trefoil: *P* = 0.001, HOAs: *P* = 0.011), except for 5 mm ocular trefoil aberration (*P* = 0.074). Additionally, rotation more than 3 degrees had significantly higher ocular spherical aberration for 3 mm PD (*P* = 0.047, Mann–Whitney *U* test). The tilt ≤ 0.1 µm for 3 mm PD and < 0.25 µm for 5 mm PD both had lower intraocular coma aberration with significance in the corresponding pupil size (3 mm PD intraocular: *P* < 0.001; and 5 mm PD intraocular: *P* < 0.001; Mann–Whitney *U* test). As for intraocular HOAs, there was a significant lower in tilt ≤ 0.25 µm for 5 mm PD (*P* = 0.001; Mann–Whitney *U* test). Tables 5 and 6 displayed the correlation between the tIOL misalignments (decentration, rotation, and tilt) and all objective optical quality for 3 mm and 5 mm PD. The results of multiple regression analysis were mostly consistent with correlation analysis (Supplementary Table S1). For a pupil diameter of 3.0 and 5.0 mm, the regression analysis showed a significant correlation between rotation and intraocular trefoil, and between tilt and intraocular coma.

**Table 5.** Correlation Analysis of the Overall IOL Decentration, Rotation, and Tilt With Optical Quality Data for 3 mm Pupil Diameter

	Decentration		Rotation		Tilt (3 mm PD)	
Parameters	*r*	*P*	*r*	*P*	*r*	*P*
AR of MTF (3 mm PD) (%)^a^	−0.104	0.600	−0.224	0.253	−0.259	0.175
SR of PSF (3 mm PD)	−0.245	0.209	−0.264	0.175	−0.048	0.806
Ocular RMS HOA (3 mm PD)	0.224	0.253	0.309	0.11	0.068	0.726
Ocular RMS coma (3 mm PD)	−0.002	0.992	0.047	0.812	0.310	0.102
Ocular RMS spherical (3 mm PD)	−0.061	0.758	0.282	0.147	0.162	0.400
Ocular RMS Trefoil (3 mm PD)	0.181	0.357	0.362	0.058	−0.029	0.811
Intraocular RMS HOA (3 mm PD)^a^	0.086	0.662	0.122	0.537	**0.549** ^b^	**0.002** ^b^
Intraocular RMS coma (3 mm PD)	−0.131	0.506	0.224	0.251	**0.743** ^b^	**< 0.01** ^b^
Intraocular RMS spherical (3 mm PD)	−0.310	0.109	0.073	0.712	−0.012	0.951
Intraocular RMS Trefoil (3 mm PD)^a^	−0.070	0.972	**0.388** ^b^	**0.041** ^b^	−0.115	0.551

AR, area ratio; HOA, higher order aberration; IOL, intraocular lens; MTF, modulation transfer function; PD, pupil diameter; PSF, point spread function; RMS, root mean square; *r*, correlation coefficient; SR, Strehl ratio.

a Spearman's correlation analysis.

b Statistically significant (*P* < 0.05).

**Table 6.** Correlation Analysis of the Overall IOL Decentration, Rotation, and Tilt With Optical Quality Data for 5 mm Pupil Diameter

	Decentration		Rotation		Tilt (5 mm)	
Parameters	*r*	*P*	*r*	*P*	*r*	*P*
AR of MTF (5 mm PD) (%)^a^	−0.081	0.681	−0.252	0.196	−0.095	0.624
SR of PSF (5 mm PD)	0.091	0.644	−0.172	0.382	0.011	0.956
Ocular RMS HOA (5 mm PD)	0.136	0.49	0.343	0.074	0.111	0.567
Ocular RMS coma (5 mm PD)	0.097	0.624	0.176	0.371	0.203	0.291
Ocular RMS spherical (5 mm PD)^a^	−0.061	0.758	0.169	0.390	−0.081	0.677
Ocular RMS Trefoil (5 mm PD)^a^	0.097	0.624	0.347	0.071	−0.073	0.708
Intraocular RMS HOA (5 mm PD)	0.052	0.794	**0.415** ^b^	**0.028** ^b^	**0.653** ^b^	**< 0.01** ^b^
Intraocular RMS coma (5 mm PD)	−0.072	0.716	0.276	0.155	**0.874** ^b^	**< 0.01** ^b^
Intraocular RMS spherical (5 mm PD)	−0.309	0.109	0.36	0.06	0.09	0.644
Intraocular RMS Trefoil (5 mm PD)	−0.034	0.864	**0.620** ^b^	**< 0.01** ^b^	0.217	0.258

AR, area ratio; HOA, higher order aberration; IOL, intraocular lens; MTF, modulation transfer function; PD, pupil diameter; PSF, point spread function; RMS, root mean square; *r*, correlation coefficient; SR, Strehl ratio.

a Spearman's correlation analysis.

b Statistically significant (*P* < 0.05).

## Discussion

Higher order aberrations, including spherical aberration, coma, and trefoil aberration, have been shown to affect retinal image quality.^15^^,^^16^ Aspherical IOL with a negative spherical aberration was introduced to compensate for the positive aspherical aberration of the cornea. This design provides benefits in terms of contrast sensitivity and enhancement of life quality.^17^ Nevertheless, HOAs in pseudophakic eyes were found to be higher than the results predicted in vitro measurements, which may be interpreted by the IOL displacement in the capsular bag.^15^^,^^18^ Thus, alterations in IOL position can impact the optical performance of IOLs.^16^^,^^19^

To the best of our knowledge, this is the first study to explore the relationship between the plate haptic bitoric IOL misalignment (including decentration, rotation, and tilt) and objective optical quality assessed by OPD-scan III in the real world. Within this study, the retroillumination images captured by OPD-scan III were used to quantify the extent of decentration and rotation. Simultaneously, the tIOL tilt was directly evaluated by the intraocular tilt aberration for 3 mm and 5 mm PD. To explore the connection between the tIOL misalignment and HOAs, the patients were compared by dividing into two groups based on a predetermined cutoff value, and the correlated analysis was conducted.

Our investigation determined that the influence of decentration on objective optical quality was minimal, with no significant differences observed between cases of decentration below and above 0.25 mm. Our findings are in agreement with a study conducted by Xu et al., indicating that optical quality data were not experience significant degradation when decentration exceeded 0.25 mm in monofocal IOLs.^20^ As previous studies reported,^21^^,^^22^ aspheric monocular IOL and EDOF IOL are unaffected by decentration up to 0.5 mm. This characteristic also accounts for the absence of significant differences identified in our study, as there were only 2 instances with more than 0.5 mm of decentration. Moreover, earlier researches have indicated that monofocal IOLs are less prone to degradation of optical quality attributed to IOL degradation when compared to multifocal IOLs.^20^^,^^22^ Tognetto et al. study demonstrated that aspheric monofocal IOL presented better MTF values with IOL decentration greater than 0.25 mm and more resistant to decentration up to 0.5 mm.^22^ Furthermore, the Zhang et al.^13^ study of investigating optical performances of tIOL decentration by a theoretical pseudophakic model eye revealed that the tolerance to decentration of tIOL was comparable to that of spherical monofocal IOLs when the astigmatic axis aligned. In order to investigate the effect of spherical aberration correction on image quality, the Pérez-Gracia et al.^23^ in vitro study using an optical bench (PMTF, Lambda-X) believed that spherical IOLs are more immune than aspherical IOLs to misalignment depending on the degree of spherical aberration correction, which can also explain why the decentration did not significantly differ in this kind of aberration-free tIOL. Nonetheless, a numerical and experimental study conducted by Pérez-Gracia et al.^24^ indicated that the coma was increasing with decentration depending on the cylinder power of tIOL. The +4.5 D cylinder power IOL in the study were only two eyes, and the significant differences between CYL ≤ +2.5 D and > +2.5 D in AR of MTF and SR of SPF were not observed. Therefore, the potential explanation of the discrepancy could be attributed to differences in tIOL model, aberration calculation, and the distribution of cylinder power of tIOL.

It has been reported that a 1 degree of a tIOL misalignment may result in an increasing residual cylinder of 3.3% of the initial power. Aside from the alteration in astigmatism correction due to tIOL rotation, aberrations can also be affected. In this study, the median rotation was recorded at 2.7 degrees, and there was significantly higher AR of MTF (3 mm and 5 mm PD) and SR of SPF (3 mm PD) and lower HOAs and trefoil aberration in the group of rotation ≤ 3 degrees compared with rotation exceeding 3 degrees. Alcocer et al. demonstrated a negative relationship between the degree of rotation and MTF in FineVision trifocal tIOLs.^25^ To study the relationship among MTF, rotation, and tilt of AcrySof toric IOL, Felipe et al.^26^ found that the average modulation for each MTF declined by roughly 50% for both 3 mm and 5 mm PD when the IOL rotated from 0 degrees to 5 degrees and was more sensitive to rotation than tilt due to smaller increments for tilt variations. These findings support the outcome of the current study, where rotations exceeding 3 degrees exhibited a significantly larger impact on MTF. In addition, they demonstrated that exponential functions fit the points representing average modulation and rotation angle satisfactorily, which can explain the low correlated coefficient in rotation and MTF in this study.^26^ Our results illustrated that HOAs and trefoil aberration significantly correlated with rotation.

No significant differences were observed in AR of MTF and SR of SPF within tilt group for 3 mm PD and 5 mm PD. Tilt exceeding 0.1 µm for 3 mm PD and 0.25 µm for 5 mm PD exhibited a notably higher level of intraocular coma in tIOL with positive correlation. In accordance with the present results, prior studies^27^ have demonstrated that intraocular HOAs were affected by normal lens tilt. To investigate the relationship between aberration measured with Scheimpflug camera and a 3-piece foldable acrylic IOL (MA30BA), Taketani et al.^19^ indicated a significant distinction related to IOL tilt and coma aberration and substantiated that minimizing IOL tilt can optimize retinal image quality.

Some limitations in the current study need to be recognized. First, the optical quality was only evaluated by objective indicators without subjective parameters, such as questionnaires. There was only one tIOL in this study without comparison with others. Further, the conclusion in this study needs to be validated by future larger patient samples.

In conclusion, this study reveals that the plate haptic tIOL was less susceptible to the optical quality degradation due to tIOL decentration while more susceptible to the intraocular trefoil aberration elevation caused by tIOL rotation, and intraocular coma aberration increase induced by tIOL tilt. The misalignment of tIOL not only leads to decline in astigmatism correction quality but also amplifies aberration, potentially compromising optical quality. However, the impact of increased aberration on visual function induced by tIOL dislocation requires further investigation.

## Supplementary Material

Supplement 1
