# Supplementary material for: Effect of Decentration, Rotation, and Tilt on Objective Optical Quality of Plate Haptic Toric Intraocular Lenses in the Early Postoperative Period
Source: Transl Vis Sci Technol. 2024 Feb 26;13(2):19. doi: 10.1167/tvst.13.2.19 (PMC10902868; doi:10.1167/tvst.13.2.19)
Supplement: Supplement 1 [file tvst-13-2-19_s001.pdf]

Table s1. Multiple regression analysis of assessing predictors of objective optical quality, including age, axial length, postoperative spherical equivalent (SE), IOL decentration, rotation, and tilt (as independent variables).

Dependent Variable	R^2 (Adjusted)	Predictor	b	β	P Value
Pupil 3.0 mm					
Ocular RMS HOA	n/s	n/s	n/s	n/s	n/s
Ocular RMS Coma	0.525	Tilt	0.197	0.737	<0.001
Ocular RMS spherical	n/s	n/s	n/s	n/s	n/s
Ocular RMS Trefoil	0.122	Rotation	0.007	0.394	0.042
intraocular RMS HOA	0.284	Tilt	0.198	0.558	0.002
intraocular RMS Coma	0.900	Tilt	0.295	0.950	<0.001
intraocular RMS spherical	n/s	n/s	n/s	n/s	n/s
intraocular RMS Trefoil	0.165	Rotation	0.004	0.444	0.020
Pupil 5.0 mm					
Ocular RMS HOA	0.145	Rotation	0.022	0.422	0.028
Ocular RMS Coma	n/s	n/s	n/s	n/s	n/s
Ocular RMS spherical	0.229	decentration	-0.168	-0.508	0.007
Ocular RMS Trefoil	n/s	n/s	n/s	n/s	n/s
intraocular RMS HOA	0.142	Tilt	0.204	0.419	0.030
intraocular RMS Coma	0.726	Tilt	0.249	0.858	<0.001
intraocular RMS spherical	n/s	n/s	n/s	n/s	n/s
intraocular RMS Trefoil	0.215	Rotation	0.014	0.496	0.009

b = unstandardized partial regression coefficient; β = standardized partial regression coefficient; IOL = intraocular lens; R^2 = coefficient of determination, adjusted for sample size
